# Concomitant Tibial Tubercle Osteotomy Decreases Odds of Revision Patellofemoral Cartilage Restorative or Palliative Surgery After Autologous Chondrocyte Implantation for Patellofemoral Cartilage Disorders

**DOI:** 10.1016/j.asmr.2025.101286

**Published:** 2025-10-17

**Authors:** Alexander R. Markes, Kyla Petrie, Alan L. Zhang, C. Benjamin Ma, Brian T. Feeley, Drew A. Lansdown

**Affiliations:** From the Department of Orthopaedic Surgery, University of California, San Francisco, San Francisco, California, U.S.A.

## Abstract

**Purpose:**

To utilize a large nationwide database to evaluate the need for revision patellofemoral cartilage restorative or palliative surgery after an index cartilage restoration procedure with and without concomitant tibial tubercle osteotomy (TTO) for patellofemoral cartilage injury.

**Methods:**

The PearlDiver Mariner Database was queried for all patients who underwent osteochondral allograft transplantation (OCA), osteochondral autograft transfer (OAT), or autologous cartilage implantation (ACI) or chondroplasty of the patellofemoral joint between 2016 and 2021 using laterality-specific International Classification of Diseases, Tenth Revision and Current Procedural Terminology codes. The 5-year revision patellofemoral cartilage restorative or palliative surgery was evaluated via χ^2^ analysis. Multivariable logistic regression was used to evaluate the association between 5-year revision patellofemoral cartilage restorative or palliative surgery and index cartilage restorative surgery with and without concomitant TTO.

**Results:**

In total, 502 patients were identified who underwent patellofemoral cartilage restorative surgery, and 61,354 patients underwent patellofemoral cartilage palliative surgery. ACI accounted for nearly half of all patellofemoral cartilage restoration procedures and increased 32% in utilization. Patients who underwent ACI were on average 4 years younger and were more likely to receive a concomitant TTO than those who underwent OCA or OAT. Patients who underwent chondroplasty were older and less likely to undergo revision cartilage restoration. The 5-year revision rates were respectively 16.6%, 13.8%, and 12.7% for ACI, OAT, and OCA, although less than 3% accounted for revision cartilage restoration. Isolated ACI had the highest odds for revision (odds ratio, 10.13; *P* < .001), although the addition of TTO attenuated those odds, with concomitant TTO and ACI having the lowest odds of revision of any procedure (odds ratio, 1.75; *P* < .001).

**Conclusions:**

Concomitant TTO with ACI for patellofemoral cartilage disorders is associated with lower odds of revision patellofemoral cartilage restorative or palliative surgery than ACI without TTO when compared to other cartilage restoration procedures.

**Level of Evidence:**

Level III, retrospective cohort study.

The patellofemoral compartment of the knee has unique anatomy. The articular cartilage of the patella is the thickest in the body, with variation in cartilage thickness across its surface, including thicker cartilage at the apex of the patella and thinner cartilage toward the periphery.^1^ The varying thickness and the unique morphology of the patellofemoral joint together create a challenging environment for surgeons to adequately address cartilage injuries.^2^ This is especially important because the patellofemoral joint also has unique wear patterns, stressors, and shear forces, all of which can cause injuries.^1^

The patella and trochlea account for 36% and 8% of all knee cartilage lesions, respectively.^3^ Previously, patients with patellofemoral cartilage defects or wear were treated primarily with tibial tubercle osteotomy (TTO) to allow for offloading of the patellofemoral articular surfaces and a decrease in contact forces.^4^ A recent meta-analysis describing TTOs in the setting of patellofemoral chondral disease showed that 78.7% had good/excellent results.^4^ Despite promising outcomes, the procedure still did not restore any cartilage and was still associated with complication rates ranging from 4.6% to 36%.^5^

More recently, procedures that aim to restore cartilage have grown in interest: autologous chondrocyte implantation (ACI), osteochondral autograft transfer (OAT), and osteochondral allograft transplant (OCA).^3^ As opposed to palliative measures, such as arthroscopic debridement (chondroplasty), these procedures looked to replace lost cartilage with predominantly type 2 collagen grafts. While studies have shown efficacy, with a systematic review indicating that 71% to 83% of patients had a significant improvement in at least 1 patient-reported outcome at a 1-year minimum follow-up, the incidence of failure still ranges from 6.8% to 22.7%.^6^ There has been increasing interest in combining osteotomy with cartilage restoration, given its ability to offload the implanted cartilage and the favorable outcomes seen in the management of condylar cartilage lesions with concomitant cartilage restoration and osteotomy.^7^ However, the role of utilizing concomitant tibial tubercle osteotomies with cartilage restoration procedures in the setting of patellofemoral cartilage restoration is still unclear.

The purpose of this study was to utilize a large nationwide database to evaluate the need for revision cartilage restorative or palliative surgery after an index cartilage restoration procedure with and without concomitant TTO for patellofemoral cartilage injury. We hypothesize that concomitant TTO will reduce the risk of revision cartilage restorative or palliative surgery following patellofemoral cartilage restoration procedures, particularly in patients undergoing autologous chondrocyte implantation.

## Methods

This retrospective cohort study was completed using PearlDiver Mariner Database (PearlDiver Technologies), a nationwide insurance billing database that provides deidentified and patient-specific claims for patients of all ages enrolled in both private-payer commercial and Medicare/Medicaid-supported insurance.^8^ The subset used for this analysis was a random subset of 157 million patients. This database allows for searching of patients with any International Classification of Diseases, Tenth Revision (ICD-10) or Current Procedural Terminology (CPT) codes and has been used in previous population-scale analyses in knee cartilage analysis and other orthopaedic surgery procedures.^9^^,^^10^

### Inclusion Criteria

All reported cases of patellofemoral cartilage restorative procedures and palliative procedures between 2016 and 2021 were included from the database using CPT codes associated with laterality-specific ICD-10 codes (Appendix Table 1, available at www.arthroscopyjournal.org) for patellofemoral joint and cartilage disorder linked to the same day. CPT codes for cartilage restorative procedures included 27412 (autologous chondrocyte implantation, knee), 29867 (arthroscopy, knee, surgical; osteochondral allograft), and 29866 (arthroscopy, knee, surgical; osteochondral autografts). CPT codes for cartilage palliative procedures included 29877 (arthroscopy, knee, surgical; debridement) and 29879 (arthroscopy, knee, surgical; abrasion arthroplasty). These cohorts were additionally queried for concomitant TTO at the time of index patellofemoral cartilage restorative or palliative procedure using the CPT code 27418 (anterior tibial tubercleplasty). Patients were excluded if they had same-day laterality-specific ICD-10 codes for chondromalacia of the knee (as opposed to the patella), primary osteoarthritis of the knee, articular cartilage tear of the knee, and osteochondritis dissecans of the knee. Articular cartilage of the knee was excluded, given the likelihood of significant overlap with cartilage restoration procedures of the femoral condyles from that given ICD code. PearlDiver also allows for “active” tracking of patients, which confirms they maintained insurance enrollment and follow-up with a provider during a specified time period. This function was used as a proxy for ensuring patients were not lost to follow-up for at least 2 years postoperatively. Additionally, use of ICD-10 coding allows for laterality-specific tracking to ensure ipsilateral revision on the same side as the index procedure. Demographic variables queried included year of surgery, patient age at time of surgery, sex, Charlson Comorbidity Index (CCI), and obesity.

### Statistical Analysis

We used *t* tests and χ^2^ tests were, respectively, to compare numerical and categorical demographic data between those who underwent patellofemoral cartilage restorative surgery and those who underwent index patellofemoral cartilage palliative surgery. Analysis of variance with a Tukey honestly significant differences post hoc test was used to compare age and CCI between a patient who underwent a specific cartilage restorative surgery and those who had other cartilage restorative surgeries. For example, this analysis allows us to directly compare the age of patients who underwent ACI compared to the age of patients who underwent either OCA or OAT. The change in annual patellofemoral cartilage restorative and palliative procedures performed from 2016 to 2021 was analyzed using multiple linear regression. Overall fit of the model was evaluated through *F* statistic, degrees of freedom, and its significance (*P* < .05). The χ^2^ analysis was used to compare the incidence of 5-year revision rates for patellofemoral cartilage restorative and palliative procedures. Descriptive analysis was used to evaluate the rate of subsequent total joint arthroplasty (TJA), as determined by procedure CPT codes (Appendix Table 1, available at www.arthroscopyjournal.org). Multivariable logistic regression was used to evaluate the association between 5-year patellofemoral cartilage revision surgery (cartilage restorative or palliative surgery) and index cartilage restoration procedure with and without concomitant TTO, as well as baseline demographics. Patellofemoral cartilage revision surgery was defined by the same CPT codes used to query for patients undergoing patellofemoral cartilage palliative or restorative surgery as described above (CPT codes 27412, 29867, 29866, 29877, 29879). All graphing analyses were performed using Excel Version 16.46 (Microsoft), while statistical analysis was performed via PearlDiver’s internal R statistical analysis tool. Significance was defined as *P* < .05. Exact *P* values were provided when able but rounded to <.001 if smaller than such values.

## Results

A total of 61,856 patients were identified who underwent patellofemoral cartilage palliative (n = 61,354) or restorative (n = 502) procedures from 2016 to 2021 and who met the inclusion criteria (Table 1). Of those 502 patients who underwent patellofemoral cartilage restoration, 51% underwent ACI, 28% OCA, and 21% OAT. Compared to those who underwent cartilage restoration, patients who underwent chondroplasty were older (age: 42.3 ± 14.5 years vs 30.9 ±10.9 years, *P* < .001), were more medically complex (CCI: 0.93 ± 1.46 vs 0.48 ± 0.74, *P* < .001), had higher rates of obesity (body mass index [BMI] >30: 48% vs 32%, *P* < .001), and were less likely to receive a concomitant TTO (4.1% vs 22%, *P* < .001). Patients who underwent ACI were on average 4 years younger than those who underwent OCA or OAT and more likely to receive a concomitant TTO at the time of the index procedure (Table 2).

**Table 1:** Patient Demographics for Patellofemoral Cartilage Restorative Surgery

Characteristic	ACI (n = 230)	OCA (n = 161)	OAT (n = 111)	*P*∗
Age, mean (SD), y *P* value†	29.7 (10.3) <.001‡	33.3 (13.8) .53	33.2 (13.3) .40	—
CCI, mean (SD) *P* value†	0.54 (0.7) .62	0.38 (1.1) <.001‡	0.52 (0.9) .31	—
Concomitant TTO	37%	8.1%	9.9%	<.001‡
Female	67%	65%	61%	.46
BMI >30	30%	35%	31%	.53

ACI, autologous chondrocyte implantation; BMI, body mass index; CCI, Charleston Comorbidity Index; OAT, osteochondral autograft; OCA, osteochondral allograft; TTO, tibial tubercle osteotomy.

∗ *P* values from χ^2^ analysis.

† *P* values from analysis of variance with Tukey honestly significant differences post hoc test.

‡ *P* < .05.

**Table 2:** Five-Year Rates of Revision Cartilage Restorative or Palliative Surgery After Index Cartilage Restorative Surgery

Characteristic	ACI	OCA	OAT	*P*
Revision patellofemoral cartilage palliative surgery	14.5%	10.7%	11.3%	.32
Revision patellofemoral cartilage restorative surgery	2.1%	2.0%	2.5%	.89

ACI, autologous chondrocyte implantation; OAT, osteochondral autograft; OCA, osteochondral allograft.

**Fig 1:**
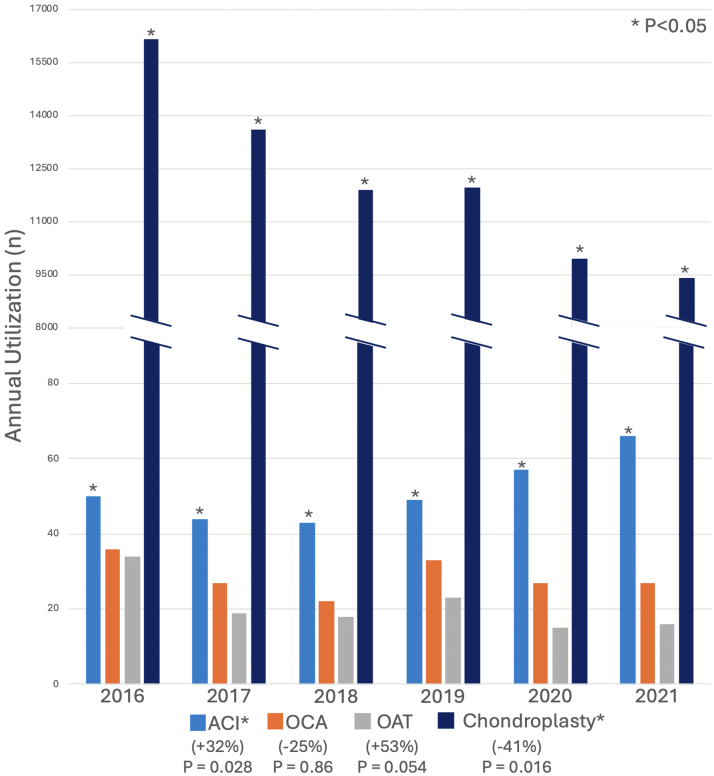
Annual incidence of patellofemoral cartilage restorative and palliative procedures. *P* < .05 is in reference to the *P* value generated from the multiple linear regression model analyzing the change in annual patellofemoral cartilage restorative and palliative procedures performed. (ACI, autologous chondrocyte implantation; OAT, osteochondral autograft; OCA, osteochondral allograft.)

ACI accounted for nearly half of all patellofemoral cartilage restoration procedures and increased 32% in utilization over the study period, while chondroplasty decreased 42% (Fig 1). The multiple linear regression model evaluating the change in annual patellofemoral cartilage restorative and palliative procedures performed showed a significant increase in ACI procedures per year (*P* = .028) and a significant decrease in chondroplasty procedures per year (*P* = .016). There were no significant changes in OCA (*P* = .86) or OAT (*P* = .054).

Five-year rates of revision cartilage restorative or palliative surgery were, respectively, 16.6%, 13.8%, and 12.7% for ACI, OAT, and OCA, with no significant differences in revision patellofemoral cartilage restorative or palliative surgery rates between groups (*P* = .29). Less than 3% of revision cartilage restorative or palliative surgery accounted for revision cartilage restoration, with 5-year rates of revision cartilage palliative surgery being 14.5%, 10.7%, and 11.3% after index ACI, OCA, and OAT, respectively (Table 2).

Patients who underwent chondroplasty were significantly less likely to undergo revision cartilage palliative or restorative procedures compared to those who underwent index cartilage restoration (5.7% vs 14.2%, *P* < .001). Of the patients who underwent an index cartilage palliative procedure, 0.9% underwent TJA 5 years from the index procedure; the incidence of TJA in patients who underwent patellofemoral cartilage restorative procedures could not be determined, given restrictions on reporting results with <10 patients inherent to PearlDiver.

In our multivariable regression analysis on odds of revision (Table 3), isolated ACI had the highest odds ratio (OR) for revision at 10.13 (*P* < .001) above OCA with an OR of 4.35 and OAT with an OR of 4.07. The addition of TTO greatly attenuated the odds of revision with ACI, as concomitant TTO and ACI had the lowest odds of revision of any procedure (OR, 1.75; *P* < .001). BMI <30, older age, male sex, and higher CCI were also protective against revision patellofemoral cartilage procedures (*P* < .001).

**Table 3:** Multivariable Regression for Odds of Revision Patellofemoral Cartilage Restorative or Palliative Surgery After Index Cartilage Restorative Surgery

Characteristic	Odds Ratio	95% Confidence Interval	*P*
ACI	10.13	9.50-10.8	<0.001∗
Concomitant TTO and OCA	8.50	7.83-9.23	<0.001∗
OCA	4.35	4.10-4.61	<0.001∗
OAT	4.07	3.85-4.30	<0.001∗
Concomitant TTO and ACI	1.75	1.69-1.81	<0.001∗
BMI >30	1.70	1.66-1.75	<0.001∗
Age	0.96	0.94-0.97	<0.001∗
Male	0.72	0.70-0.75	<0.001∗
CCI	0.69	0.68-0.70	<0.001∗
Concomitant TTO and OAT	<.01	<.01	.687

<TAB-FN>ACI, autologous chondrocyte implantation; BMI, body mass index; CCI, Charleston Comorbidity Index; OAT, osteochondral autograft; OCA, osteochondral allograft; TTO, tibial tubercle osteotomy.

∗ *P* < .05.

## Discussion

Overall, isolated ACI had the highest odds of revision among cartilage restoration procedures; however, TTO attenuated this risk, with concomitant TTO and ACI having the lowest odds of revision cartilage procedures among index cartilage restoration procedures. We observed that ACI accounted for nearly half of all patellofemoral cartilage restoration procedures and increased 32% in utilization over the study period. Chondroplasty was the preferred method of treatment for older patients, with a low 5-year rate of conversion to total joint arthroplasty.

Our data showed a high rate of concomitant TTO with ACI, along with lower revision rates when ACI was combined with concomitant TTO compared to ACI alone. Our rate of concomitant TTO with ACI (37%), OCA (8.1%), and OAT (9.9%) was very similar to the cohort in the systematic review of patellofemoral cartilage restoration done by Hinckel et al.,^6^ who found “realignment procedures” done in 38% of ACI, 6% of OCA, and 8% of OAT procedures. Overall, there are mixed data on the role of TTO on outcomes in the setting of patellar chondral injury. Concomitant TTO is thought to synergistically act to improve the outcomes after patellofemoral ACI by normalizing compressive forces and reducing abnormal shear stresses during early remodeling.^11^ One study of patients undergoing ACI, of whom 91% also underwent a TTO, showed a revision rate of 4% in their cohort.^11^ However, this rate is much lower than other studies, which cite revision rates around 15% at 5 years.^12^^,^^13^ Gomol et al.^14^ evaluated full-thickness focal cartilage lesions of the patellofemoral joint treated with ACI, in which 69% had concomitant TTO, and found that at a minimum 4-year follow-up, 84% of the patients self-rated their knees as improved. They did not find any statistically significant differences with regard to the use of TTO; however, they noted that the small control group of patients without TTO limited the strength of this subanalysis.^14^ Likewise, Burger et al.^15^ reported no difference in outcomes between patients who underwent patellofemoral cartilage restoration with or without TTO; however, they noted that the patient cohorts were too heterogeneous for adequate comparison between the two. Additionally, in our analysis, we found concomitant TTO to be associated with a higher risk of revision after OCA. We postulate that the small sample size of patients undergoing OCA or OAT with concomitant TTO for patellofemoral cartilage lesions likely contributed to this finding. Ultimately, more comparative studies must be performed to continue to elucidate indications of TTO in the treatment of patellofemoral cartilage lesions.

In our data looking at cartilage procedures in the patellofemoral joint from 2016 to 2021, we found that while rates of chondroplasty dropped, rates of ACI rose, which was the most common restorative procedure. This is consistent with Hinckel et al.,^6^ who showed in their systematic review that chondrocyte cell-based therapy (ACI) was the most commonly performed procedure, accounting for 65% of their sample. Cell-based treatment options are more amenable to fitting the variable shape of the patella and trochlea, which can be difficult to match with osteochondral procedures. This, along with technical improvements in instrumentation and scaffolding to contain the autologous chondrocytes, likely accounts for the rise in their utilization. Our study also showed that participants undergoing chondroplasty were significantly older and had a significantly higher BMI than those undergoing restorative procedures, consistent with prior literature.^9^^,^^16^ This trend of a decrease in utilization of chondroplasty, especially in younger patients, may be due to evidence that shows improved postoperative outcomes with restorative cartilage procedures compared to chondroplasty.^17^^,^^18^ Similarly, Dávila Castrodad et al.^17^ revealed improved postoperative outcomes in 98 patients with focal chondral defects of the knee when treated with cartilage-derived matrix implantation versus chondroplasty alone, with at least a 2-year follow-up. Perhaps for patients who are slightly older and less active, chondroplasty remains an acceptable option, as it has been shown to have low revision and complication rates.^16^

Our data showed 5-year revision rates of 16.6%, 13.8%, and 12.7% for ACI, OAT, and OCA, respectively. Currently, one of the longest-term follow-up studies for ACI comes from Ebert et al.,^19^ who revealed a 10-year failure rate of 10.8%. This failure rate is slightly lower than ours, but their cohort was slightly older, which may correlate with activity level and revision risk.^19^ Moreover, they defined failure via magnetic resonance imaging, TJA, or repeat ACI, whereas we defined failure via revision patellofemoral cartilage restorative or palliative procedure. Thus, our definition may overestimate true graft failure, as revision chondroplasty could involve debridement of a graft that has not entirely failed, according to the definition by Ebert et al.^19^ Moreover, Hinckel et al.^6^ performed a meta-analysis that found an overall failure rate across all procedures of 6.8% (failure was considered additional surgical procedures to revise cartilage either through restoration or arthroplasty) but a significantly higher OCA failure rate of 22.7%. However, their length of follow-up was variable across the studies.^6^

Our data also showed that age and BMI were associated with utilization of different cartilage procedures. Patients who underwent ACI were on average 4 years younger than those who underwent OCA or OAT. Moreover, in our data set, younger age, increased BMI, and female sex were all associated with an increased need for revision cartilage procedures among patients who underwent index cartilage restoration. Our findings are similar to prior research that also shows higher failure rates in younger and female patients.^11^ Female patients also had worse Lysholm and International Knee Documentation Committee scores when they had patellofemoral lesions treated with ACI.^20^ The authors report that their inclusion criteria controlled for malalignment, BMI, and age, and so they hypothesize that the sex differences in outcome scores may be due to worse proprioception and imbalances in muscle forces.^20^

### Limitations

This study is not without limitations. There is limited granularity within a publicly available database, and as such, we were unable to evaluate patient-level factors such as the location, size, or shape of the cartilage defect, alignment of the patellofemoral joint in the coronal and sagittal plane, or presence of trochlear dysplasia, which may play a role in both indications and outcomes of cartilage restoration procedures. The database is limited to the accuracy of the coding entered in the database. We have included active tracking up to a 2-year follow-up, given the limitations of the database to minimize loss to follow-up; however, we acknowledge that there remains a risk of loss to follow-up after 2 years. Although we were able to determine sex distribution as a percentage, we could not disaggregate data by sex for any further subanalysis, given the limitation in the database with lower sample sizes. We also did not extract data on other concomitant procedures besides TTO at the time of index, as there could be other variables leading to increased risks of revision. We were able to use active tracking to ensure no loss to follow-up for 2 years postoperatively. However, since we have only used ICD-10 coding for that duration, there may instances of loss to follow-up between 2 and 5 years, which can bias results.

## Conclusions

Concomitant TTO with ACI for patellofemoral cartilage disorders is associated with lower odds of revision patellofemoral cartilage restorative or palliative surgery than ACI without TTO when compared to other cartilage restoration procedures.

## Disclosures

The authors declare the following financial interests/personal relationships which may be considered as potential competing interests: A.L.Z. is a journal editor of *Arthroscopy Techniques* and a consultant or advisor for Stryker, DePuy Synthes Mitek Sports Medicine, and CONMED. C.B.M. is a consultant or advisor for Stryker and CONMED and has received a research grant from Aesculap. B.T.F. is a journal editor of *Journal of Shoulder and Elbow Surgery* and *Current Review in Musculoskeletal Medicine*. D.A.L. is a consultant or advisor for Vericel and AlloSource and has received educational support from Arthrex. All other authors (A.R.M., K.P.) declare that they have no known competing financial interests or personal relationships that could have appeared to influence the work reported in this paper.

## References

[1] Grelsamer R.P., Weinstein C.H. (2001). Applied biomechanics of the patella. Clin Orthop Relat Res.

[2] Brophy R.H., Wojahn R.D., Lamplot J.D. (2017). Cartilage restoration techniques for the patellofemoral joint. J Am Acad Orthop Surg.

[3] Shanmugaraj A., Coughlin R.P., Kuper G.N. (2019). Changing trends in the use of cartilage restoration techniques for the patellofemoral joint: A systematic review. Knee Surg Sports Traumatol Arthrosc.

[4] Rosso F., Rossi R., Cottino U., Bonasia D.E. (2022). Tibial tubercle osteotomy for patellofemoral malalignment and chondral disease provided good outcomes: A systematic review. J ISAKOS.

[5] Stokes D.J., Elrick B.P., Carpenter M.L. (2024). Tibial tubercle osteotomy: Indications, outcomes, and complications. Curr Rev Musculoskelet Med.

[6] Hinckel B.B., Pratte E.L., Baumann C.A. (2020). Patellofemoral cartilage restoration: A systematic review and meta-analysis of clinical outcomes. Am J Sports Med.

[7] Mabrouk A., An J.S., Kley K., Tapasvi K., Tapasvi S., Ollivier M. (2024). Combined knee osteotomy and cartilage procedure for varus knees: Friend or foe? A narrative review of the literature. EFORT Open Rev.

[8] Bolognesi M.P., Habermann E.B. (2022). Commercial claims data sources: PearlDiver and individual payer databases. J Bone Jt Surg.

[9] DeFroda S.F., Bokshan S.L., Yang D.S., Daniels A.H., Owens B.D. (2021). Trends in the surgical treatment of articular cartilage lesions in the United States from 2007 to 2016. J Knee Surg.

[10] Markes A.R., Cevallos N., Lansdown D.A., Ma C.B., Feeley B.T., Zhang A.L. (2022). Risk for recurrent instability and reoperation following arthroscopic and open shoulder stabilization in a large cross-sectional population. JSES Int.

[11] Zarkadis N.J., Belmont P.J., Zachilli M.A. (2018). Autologous chondrocyte implantation and tibial tubercle osteotomy for patellofemoral chondral defects: Improved pain relief and occupational outcomes among US Army servicemembers. Am J Sports Med.

[12] Leite C.B.G., Huff L.W., Medina G.I.S., Cole B.J., Lattermann C. (2023). Cartilage restoration of the patellofemoral joint: Techniques and outcomes. Oper Tech Sports Med.

[13] Athiviraham A., Boyajian H., Lindsay-Rivera K., Shi L., Khazai R. (2017). Early rates of revision of knee cartilage restoration surgery and conversion to arthroplasty within five years. Orthop J Sports Med.

[14] Gomoll A.H., Gillogly S.D., Cole B.J. (2014). Autologous chondrocyte implantation in the patella: A multicenter experience. Am J Sports Med.

[15] Burger D., Feucht M., Muench L.N., Forkel P., Imhoff A.B., Mehl J. (2022). Good clinical outcomes after patellar cartilage repair with no evidence for inferior results in complex cases with the need for additional patellofemoral realignment procedures: A systematic review. Knee Surg Sports Traumatol Arthrosc.

[16] Tuthill T., Jackson G.R., Schundler S.F. (2023). Radiofrequency chondroplasty of the knee yields excellent clinical outcomes and minimal complications: A systematic review. Arthrosc Sports Med Rehabil.

[17] Dávila Castrodad I.M., Kraeutler M.J., Fasulo S.M., Festa A., McInerney V.K., Scillia A.J. (2022). Improved outcomes with arthroscopic bone marrow aspirate concentrate and cartilage-derived matrix implantation versus chondroplasty for the treatment of focal chondral defects of the knee joint: A retrospective case series. Arthrosc Sports Med Rehabil.

[18] Jones K.J., Kelley B.V., Arshi A., McAllister D.R., Fabricant P.D. (2019). Comparative effectiveness of cartilage repair with respect to the minimal clinically important difference. Am J Sports Med.

[19] Ebert J.R., Zheng M., Fallon M., Wood D.J., Janes G.C. (2024). 10-Year prospective clinical and radiological evaluation after matrix-induced autologous chondrocyte implantation and comparison of tibiofemoral and patellofemoral graft outcomes. Am J Sports Med.

[20] Kreuz P.C., Müller S., Von Keudell A. (2013). Influence of sex on the outcome of autologous chondrocyte implantation in chondral defects of the knee. Am J Sports Med.

