# Supplementary material for: Concomitant Tibial Tubercle Osteotomy Decreases Odds of Revision Patellofemoral Cartilage Restorative or Palliative Surgery After Autologous Chondrocyte Implantation for Patellofemoral Cartilage Disorders
Source: Arthrosc Sports Med Rehabil. 2025 Oct 17;7(6):101286. doi: 10.1016/j.asmr.2025.101286 (PMC12800848; doi:10.1016/j.asmr.2025.101286)
Supplement: Appendix 1 [file mmc1.docx]

**Appendix 1**

**Current Procedural Terminology (CPT) codes of Patellofemoral Cartilage Restorative and Palliative Procedures and related procedures**

27412 - Autologous chondrocyte implantation, knee

29867 - Arthroscopy, knee, surgical; osteochondral allograft

29866 - Arthroscopy, knee, surgical; osteochondral autograft

29877 - Arthroscopy, knee, surgical; debridement

29879 - Arthroscopy, knee, surgical; abrasion arthroplasty

27418 - Anterior tibial tubercleplasty

**CPT codes for total joint arthroplasty**

27445 - Arthroplasty, knee, hinge prosthesis (eg, Walldius type)

27446 - Arthroplasty, knee, condyle and plateau; medial OR lateral compartment

27447 - Arthroplasty, knee, condyle and plateau; medial AND lateral compartments with or without patella resurfacing (total knee arthroplasty)

**ICD -10 Diagnosis codes for Patellofemoral cartilage injury**

M22.41 – Chondromalacia Patellae Right knee

M22.42 – Chondromalacia Patellae Left knee

M22.8X1 – Other disorders of Right Patella

M22.8X2 – Other disorders of Left Patella

M22.91 – Unspecified disorders of Right Patella

M22.92 – Unspecified disorders of Left Patella

M22.3X1 – Other disorders of Right Patella

M22.3X2 – Other disorders of Left Patella

**ICD -10 Exclusion codes to narrow diagnosis of patellofemoral injury**

S83.31XA – Tear of articular cartilage right knee

S83.32XA – Tear of articular cartilage Left knee

M93.261 - Osteochondritis dissecans right knee

M93.262- Osteochondritis dissecans left knee

M94.8X6- Other specified disorders of cartilage lower leg

M94.262- Chondromalacia left knee

M94.261- Chondromalacia right knee

M23.8X1- Other internal derangements of right knee

M23.8X2- Other internal derangements of left knee

M17.12- Unilateral primary osteoarthritis left knee

M17.11- Unilateral primary osteoarthritis right knee
